# Global analysis of patterns of gene expression during *Drosophila *embryogenesis

**DOI:** 10.1186/gb-2007-8-7-r145

**Published:** 2007-07-23

**Authors:** Pavel Tomancak, Benjamin P Berman, Amy Beaton, Richard Weiszmann, Elaine Kwan, Volker Hartenstein, Susan E Celniker, Gerald M Rubin

**Affiliations:** 1 Department of Molecular and Cell Biology, University of California, Berkeley, CA 94720, USA; 2 Howard Hughes Medical Institute, Cyclotron Road, Berkeley, CA 94720, USA; 3 Max Planck Institute of Molecular Cell Biology and Genetics, Pfotenhauerstr., Dresden, D-01307, Germany; 4 Department of Preventive Medicine, Keck School of Medicine of USC, Eastlake Ave, Los Angeles, CA 90033, USA; 5 Lawrence Berkeley National Laboratory, Cyclotron Road, Berkeley, CA 94720; 6 Department of Molecular Cell and Developmental Biology, University of California Los Angeles, Los Angeles, CA 90095, USA; 7 Janelia Farm Research Campus, HHMI, Helix Drive, Ashburn, VA 20147, USA

## Abstract

**Background:**

Cell and tissue specific gene expression is a defining feature of embryonic development in multi-cellular organisms. However, the range of gene expression patterns, the extent of the correlation of expression with function, and the classes of genes whose spatial expression are tightly regulated have been unclear due to the lack of an unbiased, genome-wide survey of gene expression patterns.

**Results:**

We determined and documented embryonic expression patterns for 6,003 (44%) of the 13,659 protein-coding genes identified in the *Drosophila melanogaster *genome with over 70,000 images and controlled vocabulary annotations. Individual expression patterns are extraordinarily diverse, but by supplementing qualitative *in situ *hybridization data with quantitative microarray time-course data using a hybrid clustering strategy, we identify groups of genes with similar expression. Of 4,496 genes with detectable expression in the embryo, 2,549 (57%) fall into 10 clusters representing broad expression patterns. The remaining 1,947 (43%) genes fall into 29 clusters representing restricted expression, 20% patterned as early as blastoderm, with the majority restricted to differentiated cell types, such as epithelia, nervous system, or muscle. We investigate the relationship between expression clusters and known molecular and cellular-physiological functions.

**Conclusion:**

Nearly 60% of the genes with detectable expression exhibit broad patterns reflecting quantitative rather than qualitative differences between tissues. The other 40% show tissue-restricted expression; the expression patterns of over 1,500 of these genes are documented here for the first time. Within each of these categories, we identified clusters of genes associated with particular cellular and developmental functions.

## Abstract 2

Embryonic expression patterns for 6,003 (44%) of the 13,659 protein-coding genes identified in the *Drosophila melanogaster *genome were documented, of which 40% show tissue-restricted expression.

## Background

A defining feature of multi-cellular organisms is their ability to differentially utilize the information contained in their genomes to generate morphologically and functionally specialized cell types during development. Regulation of gene expression in time and space is a major driving force of this process.

A gene's expression pattern can be defined as a series of differential accumulations of its products in subsets of cells as development progresses. Patterns of mRNA expression are studied by two principal methods - microarray analysis [1] and *in situ *hybridization [2,3]. Microarray analysis provides both a quantitative measure of gene expression and an overview of the temporal dynamics of gene expression regulation [4]. A major limitation of microarray analysis is that obtaining spatial information depends on the dissection or cell-sorting of specific tissues or cell types [5,6]. RNA *in situ *hybridization has the potential to reveal both spatial and temporal aspects of gene expression during development. However, RNA *in situ *hybridization is not quantitative [7]. For these reasons, we have used both methods in parallel and integrated the analysis of the resultant datasets.

There are several reasons for choosing *Drosophila melanogaster *as an organism for the global study of gene expression during embryonic development. Genetic and molecular analyses have led to a deep understanding of many embryonic processes in this animal [8]. Classical embryology has provided a solid framework for the anatomical description of embryonic stages [9] and robust high-throughput methods for assaying gene expression by whole mount *in situ *hybridization have been developed [10-12]. In many cases, the wild-type gene expression pattern has informed the interpretation of the phenotype produced by its mutation [13]. Such studies have provided unprecedented insights into animal development; the process that governs the early embryonic patterning of the *Drosophila *body plan is now the best understood example of a complex cascade of transcriptional regulation during development [14,15].

We have assembled an atlas of gene expression patterns during *Drosophila *embryogenesis. Taking advantage of non-redundant gene collections [16,17], we performed an unbiased survey of gene expression by using RNA *in situ *hybridization of gene specific probes to fixed *Drosophila *embryos [12] and documented the patterns with a set of digital photographs. We describe the tissue specificity of gene expression at each stage range using selected terms from a controlled vocabulary (CV) for embryo anatomy [18]. The CV integrates the spatial and temporal dimensions of the gene expression patterns by linking together intermediate tissues that develop from one another. It also integrates morphological and molecular description of development by allowing for structures that are morphologically indistinguishable and can be defined only on the basis of gene expression. We show that the genes sampled, representing 44% of the *Drosophila *genes, are largely representative of the genome as a whole, allowing the global analysis of gene expression during the embryonic development of a multicellular organism. We organized the complex gene expression space by a hybrid fuzzy-clustering approach that uses microarray profiles to supplement the CV annotation of *in situ *patterns. We divided the resulting clusters into two categories, broad and restricted. Broad patterns are characterized by quantitative enrichment in tissues that are related by specific cellular states. Restricted patterns are highly diverse and provide a basis for defining gene sets expressed in related tissues and with related predicted functions.

## Results and discussion

### Annotation dataset

The starting point for our analyses is a collection of 6,003 genes whose embryonic expression patterns we have assayed by *in situ *hybridization and systematically annotated with CVs (Release 2.0). The number of genes in the dataset has more than doubled from Release 1 [12], from 2,179 to 6,003, and the accuracy of the annotation has been significantly enhanced by performing a full re-evaluation of every gene by a second, independent curator (Materials and methods; Additional data file 1). Release 2.0, including 74,833 staged embryo images and accompanying CV annotations and microarray data, is publicly available via a searchable database [19], providing a convenient way to mine the dataset for particular expression patterns. To determine how representative our sample is, we compared the distribution of selected Gene Ontology (GO) functional annotations (generic GO slim [20]) between the 6,003 genes in our subset and the 14,586 genes in the Release 4.3 genome (Additional data file 2). No major biases for a specific molecular function, component or process were detected. Our dataset is slightly enriched for genes with known or inferred GO functions, and is, therefore, slightly deficient for genes with unknown assignment. Genes in this category lack conserved sequence features that would relate them to genes in other organisms, and may be expressed at very low levels, leading to a relative under-representation in expressed sequence tag (EST) collections. We conclude that our dataset contains a largely representative sample of gene expression patterns in the *Drosophila *genome.

**Figure 1:**
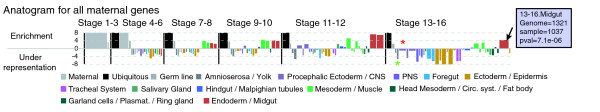
Normalized anatomical signature - the anatogram. A linear representation of the CV is used to show the enrichment of annotations within the set of all 3,334 maternally expressed genes versus the entire dataset of 4,759 genes expressed in the embryo. A vertical black line delimits stages, and each colored bar represents an individual CV term (an expanded color key is shown in Additional_data_fille 3). The width of each bar is proportional to the number of times a term was used in our entire dataset, and the height represents the relative enrichment of the given term within the particular gene set (in this case, all maternally expressed genes). Enrichment is given in units of standard deviation above or below the expected sample count based on the background frequencies (z-score). Terms with bars below the zero line are under-represented in the sample. The green asterisk corresponds to the 'amnioserosa' term, while the red asterisk corresponds to the 'brain' term. On the web supplement [21], the user can place the mouse pointer over any bar in the anatomical signature (arrow on the midgut bar in stage range 13-16) and obtain the gene count for the term in the entire dataset, the gene count within the particular set of genes under study, and a statistical *p *value of statistical over- or under-representation within the set (shown in the black bordered lavender box).

To annotate gene expression patterns, we used a set of 314 anatomical terms selected from the broad *Drosophila *Controlled Vocabulary for Anatomy maintained by FlyBase [18]. We grouped developmental structures into 16 color-coded organ systems, and reduced the full 314-term CV to 145 terms by collapsing rarely used or difficult to distinguish sub-terms to their corresponding parent term (Materials and methods; Additional data files 3-5). In order to compare the gene expression properties for a set of related genes, we created a representation of the hierarchical CV that fits on a single line, which we call an 'anatomical signature', or 'anatogram'. Figure 1 shows an anatogram for the set of 3,334 genes showing maternal expression. The relative enrichment or under-representation of CV annotations in this set of genes is indicated by the direction and height of the bar corresponding to each term, while the width of the bar indicates the genome-wide frequency of the term. Thus, commonly used annotation terms such as 'brain' (Figure 1, red asterisk) have wider bars than rare terms such as 'amnioserosa' (Figure 1, green asterisk). We used the anatomical signature to summarize groups of genes in this paper and in the accompanying supplementary online material [21].

### Organization of gene expression data using a hybrid clustering approach

Of the 6,003 genes annotated, 4,759 (79%) showed detectable expression in the embryo, while the remaining 1,244 (21%) were annotated with only the 'No staining' CV term. By grouping genes with identical annotations, the 4,759 genes with detectable expression in the embryo were subdivided into 205 multi-gene groups and 2,335 'singleton' groups (that is, groups consisting of a single uniquely annotated gene). By relaxing the criteria and grouping genes that had at least 75% of their annotation terms in common, we identified 393 multi-gene groups and 1,804 singletons. If we consider each of the multi-gene groups and each of the singleton groups to represent a distinct expression pattern, this method suggests that there are up to 2,197 distinct patterns within our dataset (Additional data file 6).

**Figure 2:**
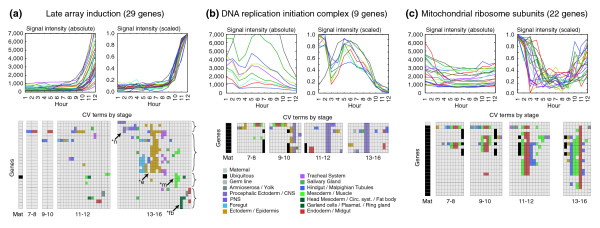
Microarray data can supplement, but not supplant, *in situ *gene expression patterns. Microarray data and the CV annotations are shown for genes **(a) **restricted to particular tissues late in embryogenesis, and **(b,c) **for broadly expressed genes encoding basic cellular protein complexes. Genes in (a) show strikingly similar array profiles but are expressed in quite diverse tissues. Late in embryogenesis half resolve to the epidermis (*e), and the other half are expressed in muscle (*m), fat body (*fb), and nervous system (*n). The genes of the DNA replication complexes, origin recognition complex and minichromosome maintenance complex display a characteristic pattern with peak expression at hour 5 (stage 10) and late expression in CNS (b). Similarly, the mitochondrial ribosomal genes decline during early embryogenesis but begin to rise around hour 10 (stage 13), with *in situ *hybridization most common in the midgut and muscle (c). For these broadly expressed gene classes the similarity of the microarray profiles is useful for supplementing the description of the *in situ *hybridization patterns using the CV annotations.

To further refine the number of expression categories, we developed a clustering strategy that allowed us to incorporate the quantitative temporal expression data obtained from the microarray experiments together with the qualitative, but spatially rich, data on expression patterns from the CV annotations. We implemented this approach within the framework of fuzzy c-means clustering [22,23] and developed a similarity metric that assigns different weights to the contribution of the microarray and annotation data (Materials and methods). Our goal was to find a proper balance between the contributions of annotation similarity versus microarray similarity to the overall similarity score. We desired a score that would minimize the contribution of microarray similarity for cases like those genes in Figure 2a, which have almost identical array profiles but incompatible annotation profiles. On the other hand, we wanted a score that would use array similarity to improve the reliability of clustering of broadly expressed genes that had similar but not identical annotation profiles, such as those in Figure 2b,c. We therefore used an asymmetric mixture function that varied the contribution of microarray data based on the similarity of the annotation data (Additional data file 7). Similarity for microarray profiles was calculated using a simple correlation metric, while similarity for *in situ *annotation profiles was calculated using a custom metric that independently weighted the contribution of each developmental stage range (Materials and methods).

The fuzzy c-means algorithm is fuzzy in that each gene is assigned to one or more clusters [24]. As multiple independent regulatory elements can drive the expression of a single gene in different tissues or at different times in development, this is a desirable property for this particular clustering problem. However, despite extensive experimentation with different clustering parameters, the large diversity of expression patterns led to clusters with ambiguous boundaries. Replication experiments using random initialization variables [25] resulted in clusters that were qualitatively similar but with numerous genes redistributed between neighboring clusters [26]. Therefore, each gene was assigned a score for each cluster, and this score was used to rank the most prototypical members of the cluster first and the most ambiguous ones last, and genes with high scores in multiple independent clusters were assigned to each cluster. This scoring allowed us to define a cutoff and determine the set of 'core' genes belonging most unambiguously to one and only one cluster (Materials and methods).

**Figure 3:**
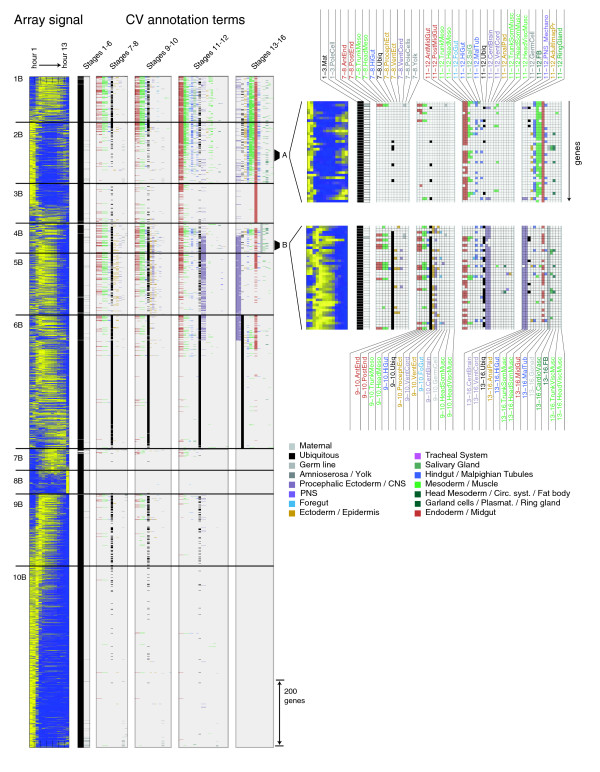
Clustered gene expression data for broadly expressed genes. We divided broadly expressed genes into 10 clusters labeled 1B-10B, each cluster separated by a horizontal black bar. From the left, we show normalized eisengrams [43] representing microarray data for 13 one-hour time points (yellow relative high expression, blue relative low expression), followed by annotation matrices split by stage range and color-coded according to organ systems. On the right is a magnified view of clusters 2B and 4B highlighting the diversity of annotations for subsets of genes.

Of 4,759 genes expressed in the embryo, we had microarray expression data for 4,496. The best fuzzy c-means run grouped these genes into 39 clusters, and each cluster was designated as either 'broad' or 'restricted'. Clusters containing a significant fraction of genes annotated as 'ubiquitous' were designated as broad, as were clusters containing primarily genes with unrestricted maternal only expression (Materials and methods). We also decided to include as broad those clusters of genes exhibiting maternal expression early and midgut-only expression late. Many genes annotated in this way (Figure 2c) encode the mitochondrial ribosomal proteins and other presumably ubiquitous mitochondrial proteins. Using these criteria, 10 of the 39 clusters (Figure 3, 1B-10B) were designated broad, and 2,549 (56.7%) genes were assigned to these clusters. The remaining 1,947 (43.3%) genes exhibited highly restricted patterns and were assigned to 29 clusters designated restricted (Table 1) [21].

**Table 1:** Division of clustering results into broad and restricted expression patterns

Clusters assigned	One	Two	Three or more	Total	Percent
No expression	1,064	0	0	1,064	19%
Broad	1,959	401	189	2,549	46%
Restricted	1,152	606	189	1,947	35%
Total	4,175	1,007	378	5,560*	100%

*Number of genes with valid microarray values for all time points. Genes assigned to both a broad cluster and any other cluster are counted only as broad.

### Broadly expressed genes

**Figure 4:**
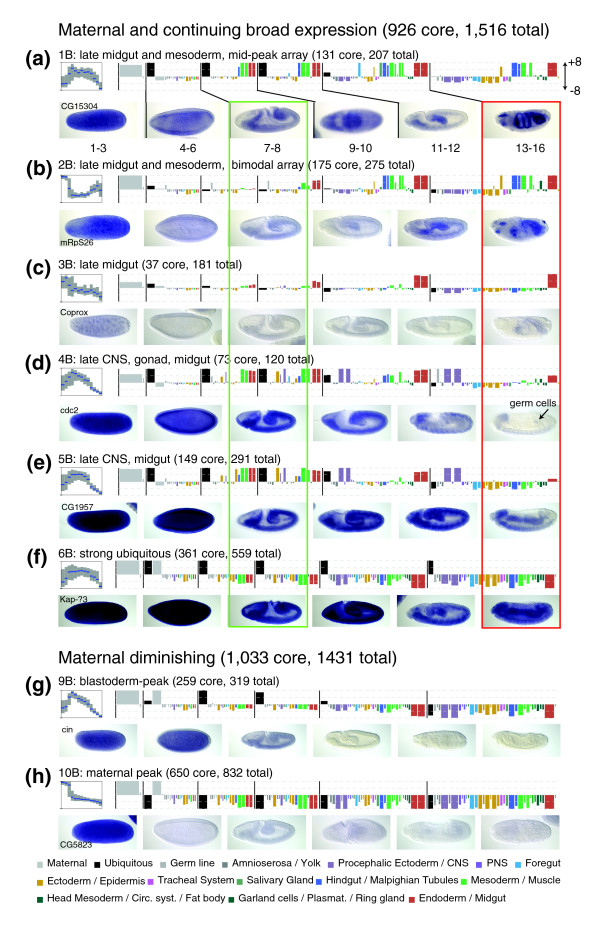
Overview of broad expression patterns. For the core genes in each broad cluster, we summarize the array profile, the annotation profile (anatogram), the number of total and core genes in the cluster and show one image for each stage of embryogenesis for a single representative gene. Array plots show the distribution of scaled intensity scores: the blue line indicates the median value while the gray box gives the inter-quartile range. The green rectangle shows that staining patterns of all broad genes are remarkably similar immediately after gastrulation. The representative late stage embryos (boxed in red) illustrate the relative diversity into which each of these homogenous early patterns resolve.

The ten clusters encompassing broadly expressed genes have relatively similar array profiles, but the diversity of annotations makes the boundaries between these clusters somewhat arbitrary (Figure 3). While there is significant ambiguity in determining the borders of these clusters, each has a distinguishing expression profile. All broad clusters (Figure 4a-h) have maternal expression followed by ubiquitous or broad expression. Genes within these clusters have stereotypical cellular functions, which reveal the physiological and cell biological states of different domains in the embryo during development.

Cluster 1B is one of the several broad clusters characterized by peak microarray expression around hours 4-5 (stage 10; Figure 4a). *In situ *hybridization showed continued ubiquitous staining throughout embryogenesis, with the heaviest staining resolving to the differentiated midgut, muscle, hindgut, foregut, and anal pads. Genes within this cluster exhibit diverse cellular functions, but within its core members are more than half of all genes known to be involved in nucleolar-based ribosome biogenesis (40 × enrichment, *p *= 5.8e-11; Additional data file 8).

Genes in cluster 2B and many in cluster 3B are characterized by peak expression levels around hour 12 (stage 15) and by *in situ *hybridization appear strongest in the differentiated midgut, muscle, hindgut, and foregut (Figure 4b,c). Cluster 2B contains 33% of all genes annotated as being mitochondrial (7 × enrichment, *p *= 2.7e-48; Additional data file 8). Genes in 3B often appear restricted to the midgut, but this cluster was classified as 'broad' due to its apparent relationship to cluster 2B, both in its overall expression profile and its enrichment for mitochondrial genes (3 × enrichment, *p *= 1.6e-5). There is a significant correlation (*p *= 3.7e-9) between the genes in clusters 2B and 3B with genes shown in an RNA interference (RNAi) screen to be induced by the histone de-acetylase SIN3, suggesting a possible regulatory mechanism [27]. A substantial fraction of these SIN3-induced genes, about 25%, are classified as having diminishing maternal staining by our *in situ *clustering (*p *= 2.6e-8 correlation with cluster 10B), suggesting that this common expression pattern is often beneath the level of detection by whole mount *in situ *hybridization.

Clusters 4B and 5B are characterized by peak expression levels around hours 4-5 (stage 10) and often resolve to exhibit staining in the differentiated nervous system and midgut (Figure 4d,e). The two clusters are differentiated by expression in the stage 13-16 gonad (Figure 4d). Both clusters are significantly enriched for genes with apparent functions in cell division, including genes required for DNA metabolism, 4B (4 × enrichment, *p *= 6.6e-5) and 5B (4 × enrichment, *p *= 5.6e-12), and the cell cycle, 4B (3 × enrichment, *p *= 4.9e-3) and 5B (4 × enrichment, *p *= 5.8e-16). Consistent with this overrepresentation of cell-cycle regulated genes, there is significant overlap between the genes in these clusters and a set of 65 genes identified in an RNAi screen for dE2F transcriptional targets [28]. We have 41 of these genes in our dataset with 40% belonging to 5B (8 × enrichment, *p *= 2.2e-12) and 20% belonging to 4B (9 × enrichment, *p *= 1.4e-6).

Genes in cluster 6B are almost uniformly annotated as ubiquitous at all stages of embryogenesis and this annotation is supported by relatively high average array expression levels at all time points (Figure 4f). Cluster 6B contains over 80% of the genes encoding the components of the cytosolic ribosome (8 × enrichment, *p *= 1.1e-29) and other genes involved in protein metabolism. Additionally, 40% of the 100 genes identified as essential for viability based on a large RNAi screen [29] are included in this cluster (4 × enrichment; *p *= 2.6e-16).

The genes in clusters 1B-6B exhibit remarkably similar expression patterns during gastrulation and were most frequently annotated as endoderm and mesoderm anlagen (Figure 4, green rectangle). This early pattern later resolves into endodermal and mesodermal derivatives for genes in clusters 1B-3B or into central nervous system (CNS) and midgut for genes in clusters 4B-5B (Figure 4, red rectangle).

Clusters 7B-10B are composed of genes with maternally deposited transcripts that diminish after stage 7 (Figure 4g,h). Those in 7B (75 genes; Figure 3) appear to rise steadily until hour 9 (stage 12), while those in 8B (49 genes) come on strongly at 16 hours (stage 16), at a time when formation of cuticle prevents efficient RNA *in situ *hybridization. Genes in cluster 9B (650 genes) show a spike in expression during the blastoderm stage, correlating with the onset of zygotic transcription, and differ from those in clusters 7B, 8B, and 10B by their annotation as 'ubiquitous' through gastrulation. It is likely that for genes in cluster 7B and 9B, the diminishing maternal expression is augmented by zygotic expression; however, a method that specifically distinguishes between maternal and zygotic transcripts is required to categorize these patterns conclusively.

The genes and expression patterns in broad clusters have largely failed to attract the attention of developmental biologists, as indicated by the fact that the embryonic expression of only 4.3% of them have been described in the scientific literature [18]. Yet, they represent more than half of the genes expressed in embryogenesis. Our analysis of broad patterns provides a comprehensive and unbiased overview of these neglected genes and redefines the definition of ubiquitous gene expression during development. A major lesson learned from our *in situ *screen is that a CV annotation strategy is insufficient to describe these patterns fully.

### Restricted expression patterns

**Figure 5:**
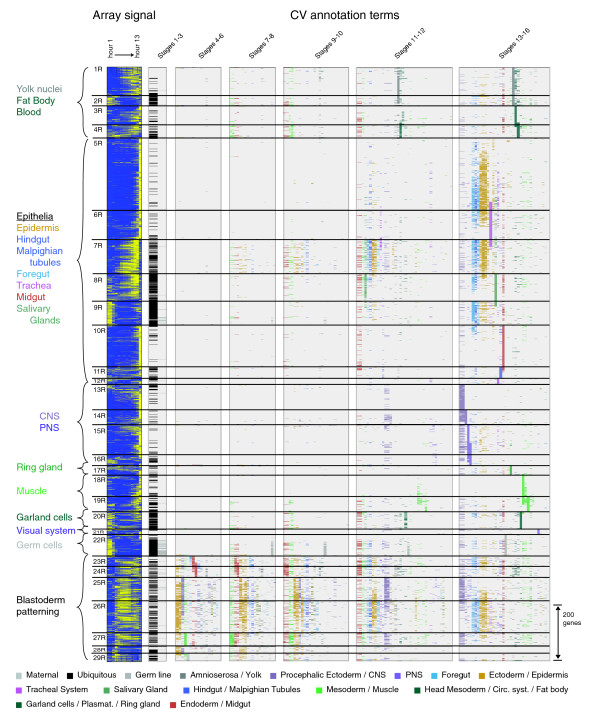
Clustered gene expression data for genes expressed in a restricted manner. We divided genes with restricted expression patterns into 29 clusters labeled 1R-29R, each cluster separated by a horizontal black bar. We used the same conventions as described for the broad clusters to capture and display the microarray and embryonic expression data (see legend to Figure 4).

While the diversity of expression patterns was considerable, our hybrid clustering approach identified a number of tissue or domain specific expression patterns shared among a significant number of genes. While these clusters are more easily categorized than the broad clusters, there is still considerable ambiguity between clusters (Figure 5).

**Figure 6:**
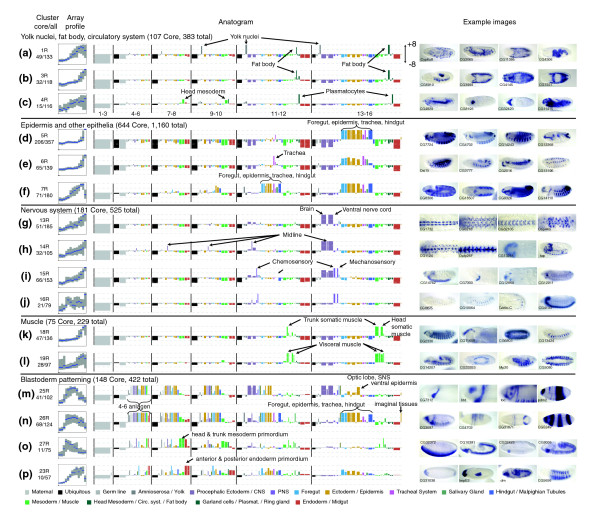
Overview of the restricted expression patterns. For unique genes in each cluster, we summarized the array profiles, diversity of annotation terms (as an anatogram), and number of total and core genes and show two to four embryo images. Whenever possible, genes with previously uncharacterized expression patterns were selected. Array plots show the distribution of scaled intensity scores: the blue line indicates the median value while the gray box gives the inter-quartile range. The most relevant annotation terms in each anatogram are labeled.

Clusters 1R-4R contain 383 genes expressed in various combinations of the yolk nuclei, fat body and blood related tissues (Figure 6a-c). Clusters 1R and 2R genes are more likely to be expressed in combinations of these different structures, while 3R genes are primarily expressed in the fat body, and 4R genes in the head mesoderm and related tissues. Interestingly, the tissues represented in these clusters derive from distinct developmental lineages, raising the question of whether a single coordinated expression program underlies expression in these seemingly unrelated developmental domains.

Clusters 5R-7R contain 1,160 genes expressed late in embryogenesis (stage range 13-16) in a number of epithelial structures (Figure 6d-f), including the epidermis, hindgut, foregut, and trachea. The epithelial pattern (Figure 6d, CG7724, CG4702) is the most recognizable and most abundant tissue-restricted pattern in embryogenesis. The epithelial expression pattern is frequently associated with expression in the tracheal system (Figure 6e). A subset of genes (Figure 6f) also showed expression in mid-embryogenesis (stages 9-12), suggesting they play a role in development and morphogenesis. The differences between the late epithelial clusters (Figure 6d,e) and the early epithelial cluster (Figure 6f) are apparent not only in the CV annotations, but also in the average microarray profiles of these clusters.

Clusters 13R-16R contain 525 genes expressed specifically in the central and peripheral nervous system (Figure 6g-j). In contrast to the genes in the broad clusters 4B and 5B that are also expressed in the nervous system, these genes lack maternally contributed transcripts and any detectable staining at or immediately after gastrulation. The CNS specific gene expression (Figure 6g) begins at stage 11 and almost always includes both the brain and the ventral nerve cord. A subset of genes (Figure 6h) is also expressed in the midline, with a small number showing transcription before stage 11. Genes expressed exclusively in the midline were extremely rare. Many genes are expressed in both the central and peripheral nervous systems (Figure 6i), while a significant number are expressed in the peripheral nervous system alone (Figure 6j).

Clusters 18R and 19R contain 229 genes expressed in either differentiated somatic muscle (Figure 6k) or differentiated visceral muscle (Figure 6l). Most genes that were detected in the visceral muscle became active earlier in the mesoderm primordia. As with the head and trunk components of the nervous system, expression in trunk muscles was almost always accompanied by expression in head muscles.

Clusters 23R-29R contain 422 genes expressed in a domain-specific manner beginning in the blastoderm stage embryo and typically continuing in a tissue-specific manner throughout embryogenesis (Figure 6m-p). Many genes are assigned to more than one cluster with only 148 (35%) assigned to a single cluster. Often genes patterned in the blastoderm show tissue-specific restricted late expression primarily in the CNS and epidermis. The relationship between blastoderm-stage expression and later tissue-specific expression is elusive. While continuity of expression in particular lineage-specific regulatory genes is well-documented, we fail to detect any statistically significant relationship between annotations at the blastoderm and later stages in our full, unbiased set of genes. While we cannot conclusively rule out that this is due to a limitation of our CV, it more likely indicates that expression of such genes is initiated independently at different stages of development rather then maintained through developmental lineages.

An additional eight clusters contain 349 genes with late tissue-specific expression (Additional data file 9a-h). Some of these contain genes expressed throughout development in a single tissue, like the cluster of genes expressed in pole and germ-cell (Additional data file 9h), while others, like the cluster of midgut-specific genes (Additional data file 9b), are primarily expressed in a particular tissue at a particular time.

**Figure 7:**
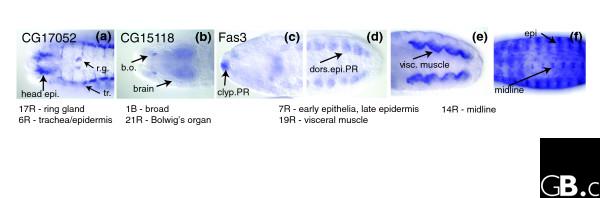
Genes classified in multiple clusters. **(a) ***CG17052 *is expressed in the ring gland as well as a number of epithelial structures at stage 14. It belongs to two clusters: 17R, the ring gland (r.g.); and 6R, the late epithelial pattern with trachea (tr.). **(b) ***CG15118 *is expressed specifically in Bolwig's organ (b.o.), along with broad staining in the brain, ventral nerve cord, anal pad, hindgut, and faintly throughout the embryo. It is classified as belonging to a broad cluster, 1B, as well as the Bolwig's organ cluster, 21R. **(c-f) ***Fas3 *has a complex expression pattern and is annotated with 27 individual annotation terms. At stage 12, it is expressed in various epithelia, including the clypeolabrum PR (clyp.PR) (c) and dorsal epidermis primordium (dorsi.epi.PR) (d), the visceral muscle PR (e) and the brain PR (not shown). At stage 15, *Fas*-*3 *is expressed in the central nervous system, including the midline, along with visceral muscle and various epithelial structures, including the trachea, hindgut, foregut, clypeolabrum, and epidermis (epi) (f). *Fas*-*3 *belongs to three clusters: 7R, the early epithelial pattern; 19R, visceral muscle; and 14R, the midline/CNS cluster.

Despite the significant number of genes that conform well to the patterns represented by the above clusters, a large fraction is expressed in unique combinations of tissues or organs. Fuzzy clustering assigned these genes to the set of clusters that best described their expression patterns. Of the 1,947 genes expressed in a restricted manner, 795 (41%) are assigned to more than one cluster (Table 1). We illustrate this by showing several examples of genes assigned to multiple clusters (Figure 7). By allowing genes to be placed into more than one expression cluster, we also hope to facilitate online searches of our dataset by representing the range of each gene's expression. The 29 restricted clusters can be viewed as distinct transcriptional programs and the numerous genes that are expressed in unique combination of tissues combine these basic programs. Such a view is consistent with our current understanding of how complex patterns of expression are generated by a set of independently acting *cis*-regulatory modules [30]. An interesting direction for future research will be to uncover the *cis*-regulatory modules that are associated with the individual restricted clusters and to examine whether or how these modules are utilized to achieve the observed diversity in gene expression.

Can we estimate the number of distinct expression patterns in *Drosophila *embryogenesis? When we use a relatively conservative measure, requiring that genes need to share 75% or more of their annotation terms to be considered 'indistinguishable', we identify 173 multi-gene groups and 1,141 singletons among the genes in our restricted clusters. Thus, by removing the broad genes, which are prone to inconsistent annotation, the number of groups within our dataset based on this measure drops from 2,197 to 1,314, providing one estimate of the number of 'distinct' patterns (Additional data file 6). On the other hand, these patterns are not unrelated. We consider the 29 restricted clusters the most prominent recurring patterns in the dataset, and we can only speculate where to place the biologically significant number of patterns within these two extremes. It is clear that the clusters are not homogenous since 41% of the genes exhibit composite patterns. If we look at all observed combinations of cluster assignments, we find 454 distinct combinations, and 287 of these cluster combinations consist of a single gene. We favor the idea that many of the composite patterns observed result from simple additive combination of the basic patterns driven by independently acting *cis*-regulatory modules. Direct examination of the patterns that each of these *cis*-regulatory modules generates in transgenic reporter assays, rather than the patterns of entire genes, will be more powerful in revealing the underlying mechanisms and logic governing the generation and evolution of each gene's expression pattern.

### Relatedness of distinct tissues

**Figure 8:**
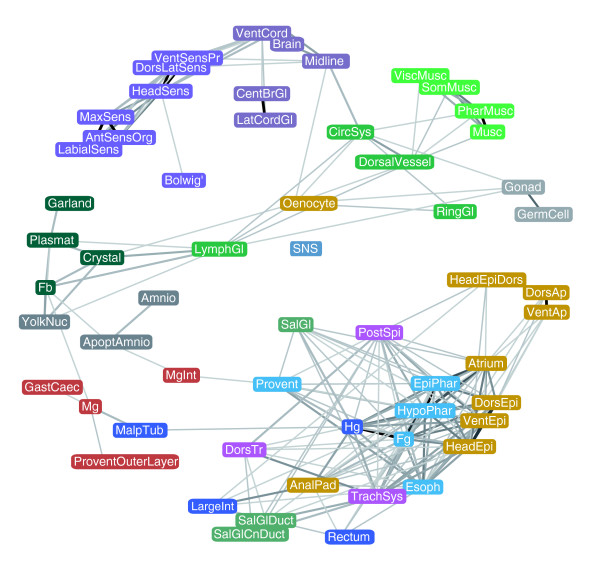
Network representation of tissue relatedness. Nodes represent collapsed annotation terms and edges represent the correlation between expression in each pair of terms. Only tissues that share a statistically significant number of genes are linked and the strength of the links is proportional to the number of genes the two tissues have in common. Tissues that share very few or no genes repel each other. The system is allowed to reach a low energy level in two-dimensional space under a physical spring model (force directed layout). Collapsed annotation terms are color-coded according to their organ system assignments as used throughout.

Besides grouping genes according to the similarity of gene expression patterns, we used our annotation dataset to define relatedness among tissues based on the similarity of the set of genes expressed in them. Figure 8 shows a network plot where tissues were connected by flexible links proportional to the fraction of commonly expressed genes and a force-directed layout was used to bring more similar tissues into proximity with each other. Tissues within individual organ systems, such as muscle (green), CNS (purple), and peripheral nervous system (violet), cluster tightly. The Bolwig's organ is isolated from the rest of the tissues, highlighting its distinct set of expressed genes. Similarly, tissues such as germ cells and amnioserosa, ring gland, stomatogastric nervous system, Malpighian tubule, midgut and garland cells share relatively few expressed genes with other tissues. In contrast, the genes expressed in the posterior spiracle, despite forming their own cluster (Additional data file 9e), appear to be components of many other tissues. As noted above, yolk nuclei, fat body and plasmatocytes share expression of a significant number of genes. In this representation, these structures are weakly related to lymph gland, which in turn shares expressed genes with the circulatory system. Many of the genes expressed in the oenocyte are also expressed in crystal cells, lymph gland, ring gland, midline, gonad and circulatory system.

The largest, most interconnected set of structures roughly corresponds to the epithelial pattern defined by clusters 5R, 6R and 7R. Notably, the salivary gland duct is isolated from the salivary gland body, reflecting their functional divergence and differential gene expression. The salivary gland duct and trachea are linked by their shared expression of genes required for cuticle deposition. In terms of gene expression, the anal pads are more similar to the hindgut than to other epidermal structures. The large distance between neural and other ectodermal derivatives suggests that specification of neuronal versus epidermal cell fate leads to profound genome-wide changes in transcription. Patterns within the digestive system are interesting - while hindgut and foregut expression are strongly correlated, midgut expression is markedly different despite its functional and spatial relatedness, reflecting its distinct developmental origin.

### Relationship between expression and function

**Figure 9:**
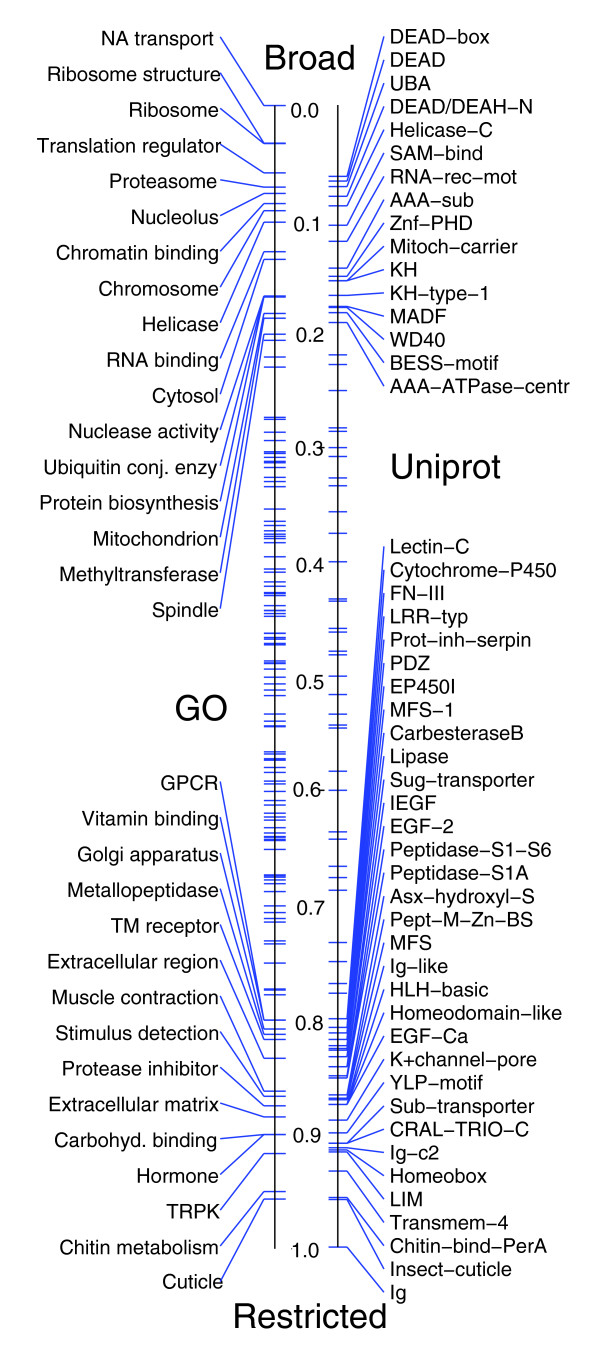
Distribution of GO annotations and Uniprot domains within broad versus restricted clusters. GO annotations (left) and Uniprot domains (right) are plotted on a number line according to the relative fraction of genes contained within broad versus restricted expression clusters. We label categories where at least 80% of the genes with patterns belong to either broad or restricted clusters.

Determining a gene's pattern of expression is a key step towards understanding its function during development. The functions of many genes have been determined, either by direct experimental analysis or by sequence homology and compiled by the GO consortium [20]. Additionally, the Uniprot database catalogs protein domains and provides phylogenetic relationships [31]. For each of our 6,003 genes, we identified associated GO terms and Uniprot domains and determined the relative distribution of these terms and domains within the broad versus restricted clusters (Figure 9), highlighting categories containing less than 20% or more than 80% restricted genes. As discussed before, broad clusters are heavily enriched for genes involved in core cellular processes, such as translation, protein degradation, cell division, energy metabolism and RNA binding proteins. The majority of transcripts for RNA binding proteins are deposited maternally into the early embryo, highlighting the necessity for mRNA processing prior to the onset of zygotic transcription. Restricted clusters are enriched in genes with sequence-specific DNA-binding domains and signaling molecules and also contain a large number of the genes involved in cuticle formation.

To examine the enrichment of GO and Uniprot categories in individual gene expression clusters, we performed exhaustive pair-wise comparisons [21]. We used the binomial test to evaluate the statistical significance of overlaps between sets of genes defined by the different data-sources. In order to correct the significance estimates for multiple testing we determined the empirical chance distribution by performing a large number of random permutations of gene functional assignments and determining the rate at which we attained particular *p *values. We interpolated these results using a log-linear regression function to fit the empirical distribution (Materials and methods). The results of this analysis are shown in Additional data file 8, which lists all GO essential (Materials and methods [21]) and Uniprot categories significantly enriched in gene expression clusters (those with an adjusted *p *value of less than 0.05 and 3-fold or greater enrichment).

**Figure 10:**
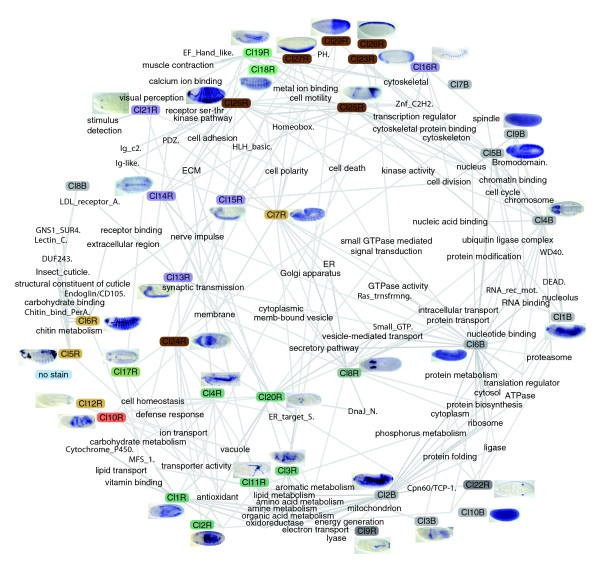
Network representation of the relationship between gene expression and gene function. Thirty-nine gene expression clusters (broad and restricted) together with the most significantly enriched GO terms and Uniprot domains (italicized) are organized in two-dimensional space by a force directed layout as in Figure 9. The strength of links between expression clusters and GO/Uniprot terms is determined by the level of enrichment of the GO/Uniprot term within the expression cluster (using z-scores in Additional data file 8). The strength of links between pairs of expression clusters and pairs of GO/Uniprot terms are determined by comparing similarity with respect to the opposite class (so that expression clusters are compared with respect to the GO/Uniprot terms they have similarity with, and vice versa; see Materials and methods). Expression cluster representative *in situ *images: Cl1B *CG12792*; Cl2B *CG4567*; Cl3B *CG407*8; Cl4B *CG2656*; Cl5B *CG3227*; Cl6B *CG7375*; Cl9B *CG8464*; Cl10B *CG13349*; Cl1R *CG3246*; Cl2R *CG8066*; Cl3R *CG2233*; Cl4R *CG4829*; Cl5R *Osi14*; Cl6R *CG32209*; Cl7R *CG12676*; Cl8R *CG14756*; Cl9R *CG10527*; Cl10R *CG1246*; Cl11R *CG633*7; Cl12R *CG9468*; Cl13R *CG15651*; Cl14R *CG31764*; Cl15R *CG14762*; Cl16R *CG18675*; Cl17R *CG8888*; Cl18R CG6429; Cl19R *CG8780*; Cl20R *CG15209*; Cl21R *CG4468*; Cl22R CG9925; Cl23R *rib*; Cl24R *CG8147*; Cl25R *CG8965*; Cl26R *odd*; Cl27R *CG12177*; Cl28R *CG13653*; Cl29R *CG1096*7.

To summarize the functional associations of gene expression clusters, we created a force-directed layout network, which brings into close proximity clusters and GO/Uniprot categories sharing a significant number of genes (Figure 10). In the force-directed layout, restricted and broad clusters separate robustly, with the notable exception of germ cell cluster 22R, which associates strongly with functions typical of broad maternal genes. This connection may be due to the fact that restriction of transcripts to the germ line lineage is often a consequence of protection of maternal message from degradation in early forming pole cells. Another cluster that violates the broad versus restricted separation is cluster 8B, which shows maternal-only expression based on *in situ *photographs but is enriched for genes involved in cuticle metabolism. Since formation of the cuticle effectively prevents RNA *in situ *hybridization, we propose that the genes in cluster 8B are likely expressed during late embryogenesis in a pattern resembling epithelial expression (similar to cluster 5R and 6R), although this pattern cannot be visualized by the standard *in situ *protocol. The late spike in the average array profile of cluster 8B genes supports this notion.

Interestingly, cluster 7R, containing genes with early (stage 12) onset epithelial expression, clearly separates from 5R and 6R, which contain genes with late epithelial expression (stages 13-16). Early epithelial expressing genes are associated with GO terms for tissue specific functions, such as membrane trafficking, morphogenesis, cell polarity, motility and adhesion, which makes them similar to genes found in the early blastoderm patterning gene cluster (cluster 26R). In contrast, late epithelial clusters (clusters 5R and 6R) associate clearly with cuticle formation in terminally differentiated tissues. This is the best example in our dataset of separation between regulatory developmental genes and effector genes [32] of the terminal cell fates.

Genes in cluster 24R are expressed in yolk, mesoderm, dorsal ectoderm and anterior and posterior endoderm anlagen at the blastoderm stage. Consistent with this early expression, these genes are expressed later in differentiated midgut, yolk, fat body and plasmatocytes. The force directed layout suggests that these genes are functionally related to clusters 1-4R, which contain genes expressed in yolk, fat body and blood and involved in metabolite transport. Cluster 24R clearly separates from other blastoderm stage clusters, suggesting that for these particular tissues, specific effector genes are required early in and throughout embryonic development.

GO terms related to membrane trafficking, such as secretory pathway, vesicle transport, Golgi apparatus, and ER, assume a central position in the layout with numerous connections to diverse clusters both broad and restricted. This likely reflects the requirement of these core cellular processes in diverse cell types, but also indicates that there are tissue specific differences in the utilization of these pathways. The modulation of these pathways is mediated by GTPases [33], which exhibit similar connectivity patterns in the force directed layout (Figure 10).

CNS and muscle clusters associate with the expected GO terms for nerve impulse transmission and muscle contraction, respectively. Interestingly, despite their clear functional specialization, both tissues show a common requirement for components of the extracellular matrix.

**Figure 11:**
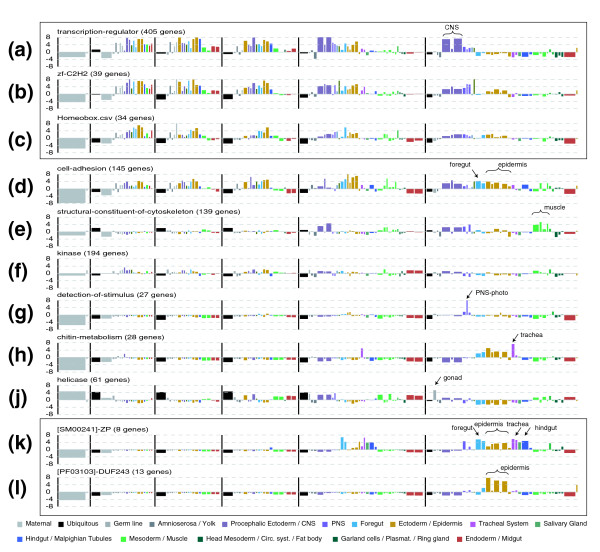
Anatogram summary for selected GO and Uniprot categories. Anatograms are used to summarize gene expression for selected **(a-j) **GO terms and **(k,l) **Uniprot protein domains. Categories related to transcriptional regulation (a-c) are boxed, as are two categories strongly enriched in clusters 5R and 6R representing epithelial patterns (k,l). Tissues discussed in the main text are labeled.

Another way to uncover relationships between gene expression and gene function is to examine the representation of GO terms in individual tissues using the 'anatograms' (Figure 11). For example, transcriptional regulators are predominantly expressed in the developing and mature nervous systems (Figure 11a). Regulation of transcription initiation by sequence-specific transcription factors is the primary mechanism used to generate tissue-specific gene expression. We determined the gene expression patterns for 238 transcription factors with sequence-specific DNA binding domains; at least one transcription factor is expressed in every tissue type recognized by our annotation hierarchy. We examined the two most abundant transcription factor classes, those with C2H2 zinc finger domains (Figure 11b) and those with homeobox domains (Figure 11c), and found that these domains show similar overall distributions, suggesting that they are deployed to regulate a similar range of developmental processes.

Cell adhesion molecules are similar to transcription factors in that they are expressed early in development in a number of anlagen, and are later abundant in the nervous system. In addition, these molecules are moderately enriched in differentiated epidermal derivatives (Figure 11d). Cytoskeletal components are enriched in the nervous system and muscles, suggesting that the tissue relatedness observed between mesodermal and neural derivatives is dictated by shared functional requirements of these cell types (Figure 11e). Interestingly, the tissue distribution of kinases is almost indistinguishable from the genome-wide average of all genes (Figure 11f). We also find strong and specific associations between genes with particular GO functions and the tissues in which they are expressed, such as stimulus and Bolwig's organ, chitin metabolism and late epithelial patterns, and helicases and gonads (Figure 11g,h,j).

Comparison of GO terms and gene expression data often leads to self-evident observations because many functional GO assignments are based on published gene expression patterns. We used the Uniprot catalog to correlate gene expression and protein domains (Figure 10). Figure 11 shows several domains expressed specifically in differentiated epidermal derivatives. For example, the zona pellucida genes encode transmembrane glycoproteins that were recently shown to be critical for tracheal morphogenesis [34]. These and other zona pellucida genes are expressed in the 5R/6R epithelial pattern (Figure 11k), which is consistent with a prior study of zona pellucida embryonic expression [35]. A novel domain that apparently exists only in flies, DUF243, is found almost exclusively in proteins encoded by genes with the late 5R pattern (Figure 11l). These tight associations of functional sequence properties and patterns of gene expression provide useful insights into how regulatory strategies are dictated by gene function.

## Conclusion

We have described the most complete set of data on spatial and temporal patterns of gene expression during embryogenesis that has been compiled for any metazoan organism. The extent, quality, and unbiased nature of this dataset allowed us to describe and explore gene expression patterns during embryogenesis on a genome wide basis. Below we discuss three issues: how this data can be used as a resource by biologists; the inherent challenges in analyzing such a complex set of data; and what we learned about global strategies for regulating gene expression during embryonic development of a complex multi-cellular organism.

### Utility of the dataset

The dataset we assembled can be used in several ways. First, it provides a rich source of candidate genes for further in-depth study. Researchers interested in a particular developmental process, for example, morphogenesis of the salivary gland, can search our annotations and retrieve a list of genes that are expressed in that structure. Such a gene set can be further subdivided by manual curation, using our primary image data. Second, the clustering classification allows one to address more abstract questions, such as: which genes are expressed in a regulated manner at cellular blastoderm? And which genes are involved in organogenesis in the late embryo? Finally, the dataset represents a starting point for an analysis of the sequence determinants of gene expression patterns. Clustering provides gene groupings based on spatio-temporal gene expression, ranging from unique patterns, through small tightly co-regulated gene sets, to large gene expression classes. These classes can be tested against *cis*-regulatory prediction pipelines to identify significant associations between gene expression specificity and genomic sequence features.

Determining expression patterns is only a first step towards further understanding gene function and, therefore, it is important to intersect our spatial expression data with other genomic datasets. Our tools allow anyone with a list of genes, for example, derived from a targeted microarray analysis, to obtain the spatio-temporal expression patterns of these genes in the *Drosophila *embryo. To address the difficulty of summarizing the gene expression patterns of a group of genes, we developed a new visual aide - the anatogram. Anatograms show the 'position' of a given gene set in the complex space of spatio-temporal gene expression patterns and represent a convenient way to summarize such data for groups of genes. Anatograms also provide an intuitive comparison of differences among groups of genes, which can supplement more rigorous statistical comparisons. Any list of genes can serve to generate an anatogram; for example, the list of *Drosophila *genes homologous to a gene group in another organism, or the genes that contain a particular sequence motif. In this way, anatograms can be used to compare results from gene expression studies among different species. The color code is based on organ systems shared by metazoan organisms and can be adapted to spatio-temporal gene expression data from other animals, providing an organism-independent way to present spatial gene expression data.

### Analysis of annotated gene expression patterns

Our results suggest that parallel microarray analysis should be an integral part of any *in situ *hybridization survey of developmental processes. Microarrays provide independent measurements that help control the artifacts of *in situ *hybridization methods, and also provide a quantitative measure of gene expression that is especially important for the interpretation of broadly expressed genes. The combined analysis of these two datasets is synergistic. *In situ *hybridization reveals the spatial diversity in tight temporal clusters and microarray clustering reduces the artificial diversity introduced by assigning annotations based on the qualitative *in situ *assay.

In the context of an anatomically well-described system such as *Drosophila *embryogenesis, it is possible to achieve great precision in expression pattern description. However, making distinctions based on the fine details of patterns, such as different subsets of the CNS, can be problematic when examining genes one by one. We found that it was useful to reduce the granularity of the CV to the level where the annotation assignments are most reliable. This approach necessarily underestimates the true diversity of expression patterns; for example, the expression of *GstS1 *in a distinct subset of cells in the midgut was annotated simply as midgut. On the other hand, this approach enables description of undefined subsets of cells and their grouping with the correct higher order structures. The fine details of differences among expression patterns on a cellular level can be addressed by comparing images of the individual members of the broader groups defined by CV annotation, or by double labeling *in situ *experiments [36,37]. A complementary approach to study gene expression of transcription factors at the blastoderm stage uses high-resolution three-dimensional confocal imaging of fluorescently labeled, fixed specimen followed by computational segmentation analysis [38,39].

Many genes are expressed ubiquitously but non-uniformly, giving the appearance of a restricted expression pattern. Identifying and correctly categorizing such ubiquitous patterns is important because their description with the standard vocabulary makes it difficult to separate them from genes with true restricted expression patterns. We identified two major classes of ubiquitous patterns, a midgut CNS pattern and an endoderm mesoderm pattern. Late in embryogenesis these differentially stained structures become apparent, whereas immediately after gastrulation there are no apparent differences among the ubiquitous patterns.

### Gene expression patterns in development

Embryonic development encompasses the complete spectrum of developmental and cell biological processes and, thus, it is not surprising that we detect the expression of 80% of the 6,003 genes we studied. Even this high number underestimates the number of genes expressed during embryogenesis. Our microarray data indicate that the late embryonic genes escaped detection in our *in situ *assay presumably because deposition of the cuticle prevents entry of the probe. In contrast, genes that are expressed in a very small subset of embryonic cells are more likely to be detected by *in situ *hybridization than by microarray analysis (data not shown).

Of genes in our unbiased set, 45% are expressed in broad patterns. Broad genes tend to encode proteins that mediate core cellular processes and their apparent patterns reflect quantitative differences in requirements for basic cellular machineries in different tissues, especially late in embryogenesis.

Of the genes in our dataset, 35% show spatially and/or temporally restricted gene expression. Our data reveal a tremendous diversity of gene expression patterns. Sets of genes that exhibit exactly the same tissue specific gene expression are rare and usually limited to mature organs. Genes with identical restricted expression patterns spanning multiple stages of embryogenesis were not found, even at the limited resolution level offered by our imaging technique. Genes that are expressed during mid-embryogenesis in a specific tissue very frequently show unrelated patterns earlier and later in development. Consequently, genes that serve as lineage markers by being expressed in a given organ system from anlagen, through primordia to final differentiated organs are rare and, for the most part, had already been discovered by genetic analysis.

In order to classify the complex expression patterns, we used a fuzzy clustering approach that allows a gene to participate in multiple clusters. We found that nearly all genes with restricted patterns fell into one of six clearly distinguishable restricted pattern types: yolk, blood and fat; epithelia; nervous system; muscle; blastoderm; or other, less frequent, organ specific patterns. Within each of these basic types, several subtypes were distinguished by their preferential expression in particular combinations of tissues.

Remarkably, 41% of the genes belong to more than one cluster, underscoring the diversity of gene expression. It is perhaps expected that the majority of gene expression patterns will be unique when one considers all developmental stages. The diversity of patterns suggests that many genes are turned on independently multiple times in development. It is less intuitive that, in terminally differentiated tissues, many genes are expressed in multiple organ systems. The existence of expression clusters indicates that the restriction of gene activities within organ systems and developmental lineages frequently occurs, whereas the fuzziness of the clusters suggests that expression in atypical combinations of tissues can be achieved. It will be interesting to investigate the *cis*-regulatory code that is responsible for initiating common patterns of gene expression and the potential for diversity in the control of gene expression. Since the control of gene expression is thought to be modular, it is possible that combinations of significantly smaller numbers of regulatory modules achieve the overall diversity of patterns.

What is the functional significance of the observed pattern diversity? Are all the minute features of the vast number of unique patterns necessary to carry out development? Or is the complexity of patterns largely a consequence of position effects in the proximity of regulatory modules that have little deleterious effect. Careful comparisons of gene expression patterns across multiple closely related species should reveal the patterns that are under evolutionary constraint. Our genome-wide dataset of patterns in *D. melanogaster *serves as a starting point for further investigation of genomic regulatory networks in development and their evolution.

## Materials and methods

### Data collection

Large-scale production of gene expression patterns by RNA *in situ *hybridization to *Drosophila *embryos was performed as described [12]. Briefly, we used digoxygenin-labeled RNA probes derived primarily from sequenced cDNAs to visualize gene expression patterns in *Drosophila *embryos by *in situ *hybridization and documented the expression patterns by digital microscopy. The histochemical color reaction was stopped in all wells of the 96-well plate at the same time once staining pattern appeared for three included control probes as well as in most wells of the plate (1-1.5 hours at 37°C). Individual embryo images were assigned to one of six stage ranges that coincide with major developmental transitions in embryogenesis, and the development of the pattern across time was confirmed with independently derived Affymetrix microarray time course data covering the first 12.5 hours of embryogenesis [19]. The images were annotated using a CV for embryonic anatomy. In the course of the primary screen, we performed 8,469 successful *in situ *experiments representing 6,580 genes. We assembled data from multiple independent experiments for 1,514 (23%) genes (labeled RNA probes were generated separately for each experiment). The same EST clone was used as the source for the probe in 1,241 of the multiple experiments, while different ESTs were used as the source in the remaining 273. Low-resolution production images were captured for all 6,003 genes. No high resolution images were captured for genes labeled as maternal, ubiquitous or no expression (2,638). At least one high-resolution image was captured for the remaining 3,365. Of these, 2,202 have high-resolution images at all six stage ranges, and 1,163 are missing at least one stage range. We captured high-resolution images only at stage ranges when a gene was expressed.

### Annotation

The primary curator (AB) assigned anatomical terms from the CV concurrently with image acquisition for each gene, providing a first pass annotation of its expression pattern. When the dataset was finalized, a second curator (VH) reviewed and edited the initial annotation assignments. In this second round of annotation, genes with similar or related patterns of expression were examined side by side; these comparisons allowed us to significantly improve the internal consistency of our annotations.

We used two approaches to review annotations (Additional data file 1). Each approach generated lists of genes with related expression patterns that were then used by the second-round curator to review the annotations of individual genes for consistency. The first approach was purely image-based and did not make use of the first round annotations. For each of the first four stage ranges, we examined images of embryos from all 6,580 genes. We developed a software tool for displaying these images in batches, and a subset of images sharing a particular feature (for example, showing regulated expression at cellular blastoderm) was manually selected. These gene lists were further subdivided until meaningful subsets could no longer be identified.

The second approach used the first round annotations to generate lists of genes ordered by similarity to particular sets of CV terms. We developed a software tool that generated lists for any arbitrary set of CV terms, but we found it most productive to define 12 relatively independent sets, each focused on a single organ system, that together covered the entire annotation hierarchy.

The lists generated by the two approaches were used to re-annotate similar genes *en masse *to make the resulting annotations as uniform as possible. CV terms were added or deleted as necessary, and genes with satisfactory and complete annotations were deemed finished and removed from all re-annotation lists. As part of this process the curator removed 577 (9%) of the genes from the dataset when the quality of the primary data was judged to be insufficient to support high-quality annotation.

### Annotation hierarchy

To describe the spatial and temporal gene expression patterns in embryogenesis, we used only two types of relationships, 'part of' to cover spatial relations and 'develops from' to cover temporal relationships among structures. Importantly, we linked terms to six developmental stage ranges and used 'develops from' relationships exclusively to link terms that belong to consecutive stage-ranges. Our anatomical terms were organized into a hierarchical tree, starting with stage range 1-3, which used only two CV terms (maternal, pole plasm), and progressively branching through the six stage ranges until stage-range 13-16 with 126 anatomical terms (Additional data file 3). Anatomical structures that were contained within a larger structure are linked to the larger structure by the 'part of' relationships (for example, 13-16 midline glia is 'part of' 13-16 midline). Anatomical structures that develop from one another across time are linked by the 'develops from' relationship (for example, 13-16 'midline' develops from 11-12 'midline primordium' (PR)). CV terms can have simultaneously the 'part of' and the 'develops from' relationships (for example, 'midline glioblast' is part of 'midline primordium', and 'midline glia' develops from the 'midline glioblast'). Every term occurs in the hierarchy only once. In a few cases where two terms develop into a single later structure (for example, 'anterior and posterior midgut primordium' forming 'midgut'), the strictly hierarchical nature of the tree is broken, and both were linked to the child term ('midgut') by the 'develops from' relationship. This fits the directed acyclic graph (DAG) format that is used to capture many biological ontologies.

Many specific structures representing small subsets of tissues had very few or none of the 6,003 interrogated genes expressed within them (50 structures had 8 genes or fewer). We summarized the data by focusing on a subset of 145 structures that make up the most common and readily distinguishable structures in our dataset. Genes annotated with more specific structures were collapsed into more general parent structures. For example, the terms 'dorsal epidermis', 'dorsal apodeme', 'dorsal histoblast nest abdominal', 'dorsal ridge' and 'leading edge cell' were collapsed into 'dorsal ectoderm'. We distinguished two levels of collapsing. Within a stage range, we collapsed the 'part of' relationships to the parent term. The resulting 'blocks' of terms represent the most relevant units of embryo anatomy for describing the RNA *in situ *results. Several such blocks may be defined within a single organ system, for example, 'trunk and head somatic and visceral musculature' (TrunkSomMusc, HeadSomMusc, TrunkViscMusc, HeadViscMusc) in the muscle system. We also collapsed terms referring to the same organ system across a range of stages: structures from stage range 4-6 were collapsed into early organ systems anlagen; structures from stage ranges 7-8 and 9-10 into mid organ systems (Additional data file 4); and structures from stage ranges 11-12 and 13-16 into late organ systems (Additional data file 5). For example, Endocrine_heart refers to the combined anatomical terms for all components of the circulatory and endocrine related structures at stages 11-16 (combining blocks 11-12 CardioVAsc, 11-12 RingGland, 13-16 CardMeso, 13-16 RingGland).

### Linear annotation profiles

Enrichment in the linear annotation profiles was displayed as the statistical significance of the over- or under-representation in the number of genes annotated with the given structure. The expected number of genes was modeled as a binomial distribution with parameters *n *(the number of genes in the list under study) and *p *(the frequency of the given structure in the dataset as a whole, $\frac{N_{s}}{4,759}$, where *N*_*s *_is the number of genes annotated with structure *s*, and 4,759 is the total number of genes expressed in the embryo). Under this model, the expected number of genes would be *np*, so gene counts greater than *np *received positive enrichment scores and counts less than *np *received negative enrichment scores. The enrichment score was simply the number of standard deviations above or below *np *in the distribution binomial (*n*, *p*), or the standard score (z-score).

### Fuzzy clustering

There were 4,496 genes detected in at least one tissue by *in situ *hybridization and present on the Affymetrix *Drosophila *1.0 gene chip. Microarray data were extracted and normalized as described in [12]. An additional time point of wild-type flies at 16 hours post egg-laying was obtained from Tiago Magalhães and normalized with the previous 12 time points using Bioconductor's RMA [40,41] package.

Input to the fuzzy clustering algorithm was the *g *× *s *binary matrix *S *of CV annotations (S for 'spatial') where *S*_*i*, *j *_is 1 when gene *i *is annotated with term *j*, and 0 otherwise; *g *is the number of genes in the dataset and *s *is the number of annotation terms. We used the 145 term collapsed version of the annotations for all clustering. An additional input matrix *L *(for 'levels') was the real-valued *g *× *t *matrix containing the normalized microarray values [40,41].

The basic procedure was similar to that outlined in [22], where [0,1] membership levels for each gene in each of *k *clusters was represented by a *g *× *k *matrix *M *and iteratively estimated. This matrix was randomly initialized with:

$$M_{ij}=\frac{1+r}{k}$$

where *r *is sampled from the uniform (0,1) distribution. The matrix was then re-normalized so that $\sum_{j=1}^{k}M_{i,j}=1$ for every gene *i*. A distance function *d*_*i*, *j *_was calculated at each iteration and used to update membership values:

$$M_{ij}=\frac{d_{ij}^{-2/(\varphi -1)}}{\sum_{l=1}^{k}d_{il}^{-2/(\varphi -1)}}$$

Here, *φ *is a 'fuzziness' parameter that determines the level of competition between clusters for the same gene (as *φ *approaches 1, it becomes a hard partition with each gene being assigned to the single best cluster, while higher values of *φ *cause gene memberships to be more fuzzy). While fuzzy clustering is generally thought to be most useful at *φ *values of 2 through 10 [24], we found that if *φ *was set above 1.5, the dataset would converge to one or two very fuzzy clusters composed of diffuse sets of terms. On the other extreme, if we used completely hard partitions (as in k-means, *φ *= 1), the majority of clusters were empty. If we used a partitioning close to 1, we found that each resulting cluster was distinct. We tried a range of values between 1 and 1.5 and used a *φ *of 1.05, which yielded the best results (data not shown).

Iterations were stopped when the average difference in the membership matrix, Δ*M *dropped below 5e^-5^, where:

$$\Delta M=\sum_{i=1}^{n}\sum_{j=1}^{k}\frac{abs(M_{ij}^{t+1}-M_{ij}^{t})}{nk}$$

We tried using classical mean [22] and medoid [42] representations for cluster centroids, but these performed poorly when attempting to combine a real-valued *L *and a binary valued *S*. Instead of maintaining a discrete centroid model to obtain distance values *d*, we instead defined *d*_*i*, *j *_as the average distance of gene *i *to all other genes *k*, where each *k *is given the weight of its membership in cluster *j*:

$$d_{ij}=\sum_{k\neq i}M_{kj}\delta_{ik}$$

Here, *δ*_*ik *_is the distance between gene *i *and gene *k*. In this way, distances between all pairs of genes are transformed into distances between genes and clusters.

### Hybrid distance function

In order to use annotation similarity to determine the contribution of array similarity (as described in Results and discussion), we defined an asymmetric mixture function where microarray similarity has a significant effect when the annotation similarity was medium to high, but very little effect when the annotation similarity was low (Additional data file 7). The mixture function defines the combined similarity *s*_*i*, *k *_in terms of the spatial similarity *s*_*s *_and the array similarity *s*_*l *_(the combined distance *δ*_*i*, *k *_is simply 1 - *s*_*i*, *k*_):

$$s_{i,k}={s^{\prime }}_{i,k}+(1-{s^{\prime }}_{i,k}){s^{\prime }}_{i,k}{s^{\prime \prime }}_{i,k}$$

The spatial similarity ${s^{\prime }}_{i,k}$ and the array similarity ${s^{\prime \prime }}_{i,k}$ were calculated separately, and were normalized to the interval [0,1] before mixing. We normalized raw similarity scores using the absolute median normalization, which is defined as:

$$x_{n}=\frac{x-v}{\sigma }$$

where *ν *is the median and the absolute deviation is:

$$\sigma =\sum_{i=1}^{n}\frac{abs(x_{i}-\nu )}{N}.$$

The microarray similarity ${s^{\prime \prime }}_{i,k}$ was simply the Pearson correlation coefficient [43]. As a measure of annotation similarity (${s^{\prime }}_{i,k}$), we have previously used the jaccard metric [12]. This metric implicitly assumed that more terms in common equates to greater similarity of expression. This is not the case for our data where the terms were related to each other in non-uniform ways, and so we designed a new metric that takes into account several important aspects of our annotation data.

Some stage ranges have only a single associated annotation term (stage range 1-3 maternal), while others have many more (stage range 13-16 has 37 collapsed terms of which up to 17 are used in a single annotation record). The relative abundance of stage range 13-16 terms dominate any metric where each term is given the same weight, so we calculated a stage range-specific similarity independently for each stage and then produced a weighted sum. Stage ranges 4-6 and 13-16 received higher weights because they coincide with two periods in embryogenesis when *de novo *transcriptional initiation most frequently occurs, cellularization and organogenesis. Stage ranges 7-8 and 9-10 represented in most cases carry-over expression from stage range 4-6, and were difficult to score and, therefore, were less reliable. The weights used for each stage range were: stage range 1-3 (7%), stage range 4-6 (36%), stage range 7-8 (7%), stage range 9-10 (7%), stage range 11-12 (7%) and stage range 13-16 (36%).

The similarity score for each stage range consisted of two components: a positive 'match bonus' score for the extent to which the two genes had terms in common, and a negative 'mismatch penalty' score for the extent to which the two genes had mismatched terms. The match bonus contributed twice as much as the mismatch penalty to the overall score:

$${s^{\prime }}_{i,k}=\sum_{r\in stages}\lambda_{r}\left(2{s^{\prime }}_{i,k}^{+}-{s^{\prime }}_{i,k}^{-}\right)$$

Where ${s^{\prime }}_{i,k}^{+}$ is the match and ${s^{\prime }}_{i,k}^{-}$ is the mismatch. Match and mismatch scores are defined as follows. Genes not sharing annotation terms at a given stage range receive a match score of 0. Genes sharing any annotation term receive a match bonus equal to 1 plus a rarity factor from 0 to 0.5. The rarity factor is inversely proportional to the abundance (amongst all annotations) of the most rare term that is shared between the two genes. The mismatch penalty is equal to 1 minus a rarity factor from 0 to 0.5. Genes sharing rare terms receive the highest overall similarity scores, and genes mismatched for rare terms receive the lowest. This has the desirable effect of keeping those genes with rare sets of terms in common more tightly clustered.

### Broad versus restricted cluster designation

The classification of clusters as broad or restricted was made automatically based on the average annotation terms of the genes assigned to the cluster. We classified a cluster as broad if any of the following criteria were met: over 30% of the genes in the cluster were annotated as ubiquitous at some stage from 7 to 16; if the cluster had 75% maternal genes and no restricted annotation terms in two-thirds of the genes; if the cluster contained genes with only maternal and late midgut staining (see Results and discussion). All other clusters were classified as restricted.

### Assignment of genes to multiple clusters

As described in the text, we did not attain a fuzzy c-means clustering result where clusters had sharp boundaries. Therefore, the raw membership matrix *M *often assigned a gene high membership scores in neighboring, highly related clusters. In order to mitigate this fact and assign multiple memberships in a meaningful way, we performed an exhaustive analysis of cluster similarities to assign each gene to a set of significantly unrelated clusters. First, the raw membership matrix was transformed to a binary matrix M* using a threshold of *m*_min_. Next, a distance score Δ_*i*, *j *_between each pair of clusters *i *and *j *was determined from M* by dividing the number of genes in common by the number in the smaller of the two clusters:

$$\Delta_{i,j}=1-\frac{\sum_{g=1}^{N_{genes}}\left(M_{g,i}^{*}M_{g,j}^{*}\right)}{\min\left(\sum_{g=1}^{N_{genes}}M_{g,i}^{*},\sum_{g=1}^{N_{genes}}M_{g,j}^{*}\right)}$$

Next, each gene was assigned to a set of dissimilar clusters using a greedy approach. The gene *g *is first assigned to the cluster *i*_*new *_with the highest membership score, that is, $\underset{k\in clusters}{\arg\max}\left(M_{g,k}\right)$. Then, all clusters *j *that are within a maximum distance *d*_max _of *i*_*new *_(that is, $\Delta_{i_{new},j}\leq d_{\max}$) are excluded from further consideration. We then go to the cluster with the next highest membership score, and the gene is successively assigned to clusters in this way until all clusters with *M*_*g*, *i *_≥ *m*_min _have been considered.

Rather than determining a single set of *m*_min _and *d*_max _values, we performed this procedure 10,000 times with all pairwise combinations of values within 0.15 ≤ *m*_min _≤ 1.0 and 0.7 ≤ *d*_max _≤ 1.0 (These ranges were chosen to sample the entire informative range of the space: *m*_min _values below 0.15 produced trivial results where each gene was only assigned to its single best cluster; *d*_max _values below 0.7 resulted in the majority of genes being assigned to multiple, highly similar, clusters.) We averaged the results across all runs, calculating the number of times a particular gene was assigned to a particular cluster. By plotting the distribution of values, we identified a natural cutoff at 400/10,000 - gene/cluster assignments occurring in at least 400 of the 10,000 runs became final.

### Force-directed layout

The 'balls and springs' models presented in Figures 9 and 11 were generated using the Prefuse package [44]. In Figure 9, we used a force-directed layout where the strength of edges between annotation terms (modeled as springs) is proportional to the jaccard similarity score:

$$s=\frac{n_{a\cap b}}{n_{a\cup b}}$$

where *n*_*a*∩*b *_is the number of genes shared by CV term *a *and CV term *b*, and *n*_*a*∪*b *_is the number of genes in either *a *or *b*. For figure 11, there are three types of edges: type I edges between GO/Uniprot terms and expression clusters; type II edges between GO/Uniprot terms and other GO/Uniprot terms; and type III edges between expression clusters and other expression clusters. Type I edges are set to the corrected standard score (z-score) enrichment statistic as described in 'Statistical comparison of large genomic datasets'. These enrichment z-scores are then used to calculate type II and type III edges. The type II edge between GO/Uniprot term *a *and GO/Uniprot term *b *is proportional to the Pearson correlation coefficient obtained when comparing the enrichment z-scores of *a *to those of *b*. In this way, type II edges are not an indication of the actual number of genes in common between two GO/Uniprot categories, but rather the extent of similarity of the 'expression patterns' of those genes. Likewise, the type III edge between expression cluster *c *and expression cluster *d *is not determined by the number of genes shared between those two clusters, but rather the similarity in function between those two sets of genes.

### Statistical comparison of large genomics datasets

We used the GO and Uniprot databases from April 2006 for all comparisons. A gene was considered to belong to a particular GO category if it associated with the category itself or any descendant of the category linked via IS_A and/or PART_OF relationships in the GO DAG. For instance, if a gene were annotated as an endopeptidase (GO:0004175), it would also be considered a peptidase (GO:0008233). Because many related GO terms have highly redundant sets of genes assigned to them, we also selected a set 179 of GO terms (which we call GO essential slim [21]) and propagated genes through the DAG to these terms using the map2slim.pl tool. A gene list was created for each GO category, GO essential category and each Uniprot domain.

To evaluate whether a given expression cluster was correlated with a particular functional category, we used a statistical binomial test. The background frequency for the functional category, *q*, was defined as the fraction of the 6,003 annotated genes assigned to the category. For an expression cluster containing *n *genes total, the background was modeled as a binomial (*n*, *q*) distribution. When the expression cluster contained *x *genes within the particular functional category, the exact probability of observing this many or more genes, Pr(*X *= *x*)~binomial (*n*, *q*), was calculated using Matlab's binocdf function (The MathWorks, Natick, MA).

We corrected the statistical significance values for multiple testing by creating an empirical distribution of binomial test scores for a large number of randomized gene lists. To generate the gene lists, genes were randomly sampled (without replacement) from the entire genome of 14,586 genes (Release 4.3). We sampled 100,000 such lists for each list size from five genes to 1,000 genes (in increments of 5 genes). Each sample was then statistically tested against all GO and Uniprot categories using the binomial test as described above. The most significant (lowest) *p *value attained (*p*_*α*_) was determined within each of the four functional classes (GO process, GO component, GO function, Uniprot domain), and the empirical distribution was compiled based on the distribution of *p*_*α *_values obtained.

In order to interpolate this empirical background distribution to any given *p*_*α *_value obtained for an actual test, we fit the distribution to a log curve using log-linear regression (Matlab glmval function), fitting the parameters *a *and *b *in the function log(*p*) = *a*log(*p*_*α*_) + *b*. This produced a function that was a good fit for all list sizes, whereas fitting a linear function (*p *= *ap*_*α *_+ *b*) generally produced fits that deteriorated at low *p *values.

## Additional data files

The following additional data are available with the online version of this paper. Additional data file 1 is a Re-annotation flowchart. The schematic of our image-driven curation strategy is shown on the left. The curator generated lists of genes (either all genes at a stage range or subset based on annotation query) and the computer produced all the images associated with those genes. Images were presented in batches that were navigated in a manner similar to Google searches with progress tracking. Pages that were viewed were marked. Gene names were shown in the corner of the first image for that gene. The curator reviewed the annotations of all genes expressed in a tissue, for example CNS, and by clicking on an image showing CNS staining, moved that image and the associated gene name to a child gene list. The images for the selected genes were eliminated from the parent pages. Both the parent and child gene lists were stored in a specialized database called the 'list manager' [21]. Lists became inputs for further image-based sub-selection or were used as a starting point for the annotation driven curation approach. The schematic of our annotation driven curation strategy is shown on the right. The curator inputs a gene list that was based on an annotation query or based on the previously described image driven curation. The curator then selected subsets of annotation terms called the 'block'. The list was ordered according to the content of block terms in the annotations of each gene. The curator was presented with images and block-limited annotations for each gene in the list in succession and changed any annotations within the block that needed correction. The curator was then presented with the set of genes that were not annotated for block terms and corrected any omissions. Modifications to the annotations were immediately stored in the database and a history of all changes was tracked. Additional data file 2 is a comparison of the distribution of selected Gene Ontology terms (GO slim general [20]) in the 6,003 genes in our study (red bars) and the distribution of the same terms in the 14,586 genes (purple bars) in the genome (Release 4.3). Each bar represents the percentage of genes with a given GO slim category. The similarities of these two profiles suggest we have annotated a representative sample of all genes. Additional data file 3 is a Description of the annotation hierarchy. We collapsed the 16 well-defined embryonic stages [9] into six developmental stage ranges. Annotation terms were grouped into the six developmental stage ranges, which are separated by solid horizontal lines. We reduced 314 terms in the full anatomical CV to 145 terms representing the structures that were most frequently seen and most readily distinguishable in our dataset, and the 131 of these that are annotated in 10 or more genes are shown here (Additional data files 4 and 5 list all terms). Genes annotated using more specific annotation terms were collapsed to this level. For example, an annotation of 'dorsal ridge' was collapsed to 'dorsal ectoderm', because the 'dorsal ridge' is 'part of' the 'dorsal ectoderm' in our formal CV hierarchy. Relative annotation counts are shown as colored bars. The length of each bar is proportional to the number of genes annotated with that term. Each bar is in one of 16 colors indicating the particular cell fate of the lineage; for instance, endoderm structures are in red across all developmental stages. This color code follows to the 'develops from' relationship among terms in our formal annotation hierarchy. Additional data files 4 and 5 contain the raw gene counts using the same organization. Additional data file 4 is a summary by organ systems and collapsed annotation terms for early embryogenesis (stages 4-10). For each organ system, we listed: the 'total' number of genes annotated with any raw annotation term that belonged to that organ system; the number of genes restricted ('restr.') to that organ system, meaning that at the stage range indicated the gene was annotated ONLY with raw terms of that organ system; the number of genes expressed in the organ system and at the same time not expressed in an equivalent organ system at another stage of development (restr. 4-6 and restr. 7-10); the total number of genes expressed in the equivalent organ systems connected by 'develops from' relationship at stages 4-6 OR stages 7-10 (st 4-10 uni. = union); and the number of genes expressed in equivalent organ system connected by 'develops from' relationship at stages 4-6 AND stages 7-10 (stages 4-10 intrs. = intersection). For each collapsed annotation term (block) we listed: the total number of genes annotated with any raw annotation term that belongs to that block; and the number of genes restricted ('restr.') to the block, meaning that at the given stage range the gene is annotated ONLY with raw terms from the block and no other terms from another block. The organ systems and blocks are color-coded. Mapping of organ systems to blocks of terms and blocks of terms to raw terms is available as supplementary on-line material [21]. Additional data file 5 is a Summary by organ systems and collapsed annotation terms for late embryogenesis (stages 11-16). Similar to Additional data file 4, we listed for each organ system and each block of terms: the total number of genes that were annotated with ANY raw annotation term that belonged to that organ system or block; the number of genes expressed ONLY in the organ system or block at the given stage (restr.); the number of genes expressed in the organ system or block and at the same time not expressed in an equivalent organ system or block at another stage of development (restr. 11-12 and restr. 13-16); the number of genes expressed in equivalent organ systems or blocks at stage range 11-12 OR stage range 13-16 (st 11-16 uni. = union); and the number of genes expressed in equivalent organ systems at stage range 11-12 AND stage range 13-16 (stages 11-16 intrs. = intersection). The organ systems and blocks are color-coded. Mapping of organ systems to blocks of terms and blocks of terms to raw terms is available as supplementary on-line material [21]. Additional data file 6 shows the diversity of CV annotations. Genes that share between 50% and 100% of their annotation terms (x-axis; uniformity) were grouped and the resulting number of groups enumerated (y-axis). The solid line in each graph shows the total number of groups thus formed for a given level of uniformity. The dashed line shows the total number of groups with a single gene member (singletons) for a given level of uniformity. For uniformity levels of 100% (all annotation terms matched within the group) and 75% (3 out of 4 terms matched within the group) we highlight the total number of groups, the number of singletons and the difference between the two. (A) Data for all genes expressed in the embryo (B) for genes belonging to broad clusters and (C) for genes in restricted clusters. Additional data file 7 shows the asymmetric similarity mixture. Given an annotation (spatial) similarity score 0 ≤ *s*_*s *_≤ 1 and an array (level) similarity score 0 ≤ *s*_*l *_≤ 1, the function *s*_*c *_= *s*_*l *_+ (1 - *s*_*l*_)*s*_*l *_*s*_*s *_gives a similarity score where microarray similarity has a significant effect when annotation similarity is medium to high, but very little effect when annotation similarity is low.

Additional data file 8 is a Complete list of GO-essential terms and Uniprot domains enriched with respect to 39 clusters (both broad and restricted) formed by hybrid clustering of gene expression data. *Terms with adjusted *p *value smaller than 0.001. B = broad, R = restricted. ^‡^Total number of unique genes in cluster. ^§^GO or Uniprot id. ^¶^GO or Uniprot name. ^¥^Ratio between observed and expected number of genes in intersection of the two gene lists. ^#^Number of genes in cluster annotated with given GO or Uniprot term. **Number of genes in the genome annotated with given GO or Uniprot term. ^tt^Adjusted *p *value of binomial z test testing the null hypothesis that the overlap between the two gene lists is random. Additional data file 9 is an overview of the remaining miscellaneous restricted expression patterns. Similar to Figure 6 for unique genes in each cluster, we summarized the array profiles, diversity of annotation terms (as an anatogram), number of total and core genes and show two to four embryo images. Whenever possible, genes with previously uncharacterized expression patterns were selected. Array plots show the distribution of scaled intensity scores: the blue line indicates the median value while the gray box gives the inter-quartile range. The most relevant annotation terms in each anatogram are labeled.

## Supplementary Material

Additional data file 1 The schematic of our image-driven curation strategy is shown on the left. The curator generated lists of genes (either all genes at a stage range or subset based on annotation query) and the computer produced all the images associated with those genes. Images were presented in batches that were navigated in a manner similar to Google searches with progress tracking. Pages that were viewed were marked. Gene names were shown in the corner of the first image for that gene. The curator reviewed the annotations of all genes expressed in a tissue, for example CNS, and by clicking on an image showing CNS staining, moved that image and the associated gene name to a child gene list. The images for the selected genes were eliminated from the parent pages. Both the parent and child gene lists were stored in a specialized database called the 'list manager' [21]. Lists became inputs for further image-based sub-selection or were used as a starting point for the annotation driven curation approach. The schematic of our annotation driven curation strategy is shown on the right. The curator inputs a gene list that was based on an annotation query or based on the previously described image driven curation. The curator then selected subsets of annotation terms called the 'block'. The list was ordered according to the content of block terms in the annotations of each gene. The curator was presented with images and block-limited annotations for each gene in the list in succession and changed any annotations within the block that needed correction. The curator was then presented with the set of genes that were not annotated for block terms and corrected any omissions. Modifications to the annotations were immediately stored in the database and a history of all changes was tracked. Click here for file

Additional data file 2 Comparison of the distribution of selected GO terms (GO slim general [20]) in the 6,003 genes in our study (red bars) and the distribution of the same terms in the 14,586 genes (purple bars) in the genome (Release 4.3). Each bar represents the percentage of genes with a given GO slim category. The similarities of these two profiles suggest we have annotated a representative sample of all genes. Click here for file

Additional data file 3 We collapsed the 16 well-defined embryonic stages [9] into six developmental stage ranges. Annotation terms were grouped into the six developmental stage ranges, which are separated by solid horizontal lines. We reduced 314 terms in the full anatomical CV to 145 terms representing the structures that were most frequently seen and most readily distinguishable in our dataset, and the 131 of these that are annotated in 10 or more genes are shown here (Additional data files 4 and 5 list all terms). Genes annotated using more specific annotation terms were collapsed to this level. For example, an annotation of 'dorsal ridge' was collapsed to 'dorsal ectoderm', because the 'dorsal ridge' is 'part of' the 'dorsal ectoderm' in our formal CV hierarchy. Relative annotation counts are shown as colored bars. The length of each bar is proportional to the number of genes annotated with that term. Each bar is in one of 16 colors indicating the particular cell fate of the lineage; for instance, endoderm structures are in red across all developmental stages. This color code follows to the 'develops from' relationship among terms in our formal annotation hierarchy. Additional data files 4 and 5 contain the raw gene counts using the same organization. Click here for file

Additional data file 4 For each organ system, we listed: the 'total' number of genes annotated with any raw annotation term that belonged to that organ system; the number of genes restricted ('restr.') to that organ system, meaning that at the stage range indicated the gene was annotated ONLY with raw terms of that organ system; the number of genes expressed in the organ system and at the same time not expressed in an equivalent organ system at another stage of development (restr. 4-6 and restr. 7-10); the total number of genes expressed in the equivalent organ systems connected by 'develops from' relationship at stage range 4-6 OR stage range 7-10 (st 4-10 uni. = union); and the number of genes expressed in equivalent organ system connected by 'develops from' relationship at stage range 4-6 AND stage range 7-10 (stages 4-10 intrs. = intersection). For each collapsed annotation term (block) we listed: the total number of genes annotated with any raw annotation term that belongs to that block; and the number of genes restricted ('restr.') to the block, meaning that at the given stage range the gene is annotated ONLY with raw terms from the block and no other terms from another block. The organ systems and blocks are color-coded. Mapping of organ systems to blocks of terms and blocks of terms to raw terms is available as supplementary on-line material [21]. Click here for file

Additional data file 5 Similar to Additional data file 4, we listed for each organ system and each block of terms: the total number of genes that were annotated with ANY raw annotation term that belonged to that organ system or block; the number of genes expressed ONLY in the organ system or block at the given stage (restr.); the number of genes expressed in the organ system or block and at the same time not expressed in an equivalent organ system or block at another stage of development (restr. 11-12 and restr. 13-16); the number of genes expressed in equivalent organ systems or blocks at stage range 11-12 OR stage range 13-16 (st 11-16 uni. = union); and the number of genes expressed in equivalent organ systems at stage range 11-12 AND stage range 13-16 (stages 11-16 intrs. = intersection). The organ systems and blocks are color-coded. Mapping of organ systems to blocks of terms and blocks of terms to raw terms is available as supplementary on-line material [21]. Click here for file

Additional data file 6 Genes that share between 50% and 100% of their annotation terms (x-axis; uniformity) were grouped and the resulting number of groups enumerated (y-axis). The solid line in each graph shows the total number of groups thus formed for a given level of uniformity. The dashed line shows the total number of groups with a single gene member (singletons) for a given level of uniformity. For uniformity levels of 100% (all annotation terms matched within the group) and 75% (3 out of 4 terms matched within the group) we highlight the total number of groups, the number of singletons and the difference between the two. (A) Data for all genes expressed in the embryo (B) for genes belonging to broad clusters and (C) for genes in restricted clusters. Click here for file

Additional data file 7 Given an annotation (spatial) similarity score 0 ≤ *s*_*s *_≤ 1 and an array (level) similarity score 0 ≤ *s*_*l *_≤ 1, the function *s*_*c *_= *s*_*l *_+ (1 - *s*_*l*_)*s*_*l *_*s*_*s *_gives a similarity score where microarray similarity has a significant effect when annotation similarity is medium to high, but very little effect when annotation similarity is low. Click here for file

Additional data file 8 *Terms with adjusted *p *value smaller than 0.001. B = broad, R = restricted. ^‡^Total number of unique genes in cluster. ^§^GO or Uniprot id. ^¶^GO or Uniprot name. ^¥^Ratio between observed and expected number of genes in intersection of the two gene lists. ^#^Number of genes in cluster annotated with given GO or Uniprot term. **Number of genes in the genome annotated with given GO or Uniprot term. ^tt^Adjusted *p *value of binomial z test testing the null hypothesis that the overlap between the two gene lists is random. Click here for file

Additional data file 9 Similar to Figure 6 for unique genes in each cluster, we summarized the array profiles, diversity of annotation terms (as an anatogram), number of total and core genes and show two to four embryo images. Whenever possible, genes with previously uncharacterized expression patterns were selected. Array plots show the distribution of scaled intensity scores: the blue line indicates the median value while the gray box gives the inter-quartile range. The most relevant annotation terms in each anatogram are labeled. Click here for file
