# Supplementary material for: Global analysis of patterns of gene expression during Drosophila embryogenesis
Source: Genome Biol. 2007 Jul 23;8(7):R145. doi: 10.1186/gb-2007-8-7-r145 (PMC2323238; doi:10.1186/gb-2007-8-7-r145)
Supplement: Additional data file 7 — Given an annotation (spatial) similarity score 0 ≤ ss ≤ 1 and an array (level) similarity score 0 ≤ sl ≤ 1, the function sc = sl + (1 - sl)sl ss gives a similarity score where microarray similarity has a significant effect when annotation similarity is medium to high, but very little effect when annotation similarity is low. [file gb-2007-8-7-r145-S7.pdf]

Pattern*	Size‡	Funcid§	Function¶	Enr¥	with pat.#
